# Analysis of cytosine-adenine repeats in P1 promoter region of IGF-1 gene in peripheral blood cells and cervical tissue samples of females with cervical intraepithelial lesions and squamous cervical cancer

**DOI:** 10.3892/mmr.2014.2916

**Published:** 2014-11-10

**Authors:** WOJCIECH KWASNIEWSKI, ANNA GOZDZICKA-JOZEFIAK, MARIA KOTARSKA, GRZEGORZ POLAK, BARTLOMIEJ BARCZYNSKI, JUSTYNA BRONIARCZYK, WITOLD NOWAK, MARIA WOLUN-CHOLEWA, ANNA KWASNIEWSKA, JAN KOTARSKI

**Affiliations:** 1 Department of Gynecological Oncology and Gynecology, Medical University of Lublin, Lublin 20-081, Poland; 2 Department of Molecular Virology, Adam Mickiewicz University, Poznan 61-614, Poland; 3 Rush Medical College, Rush Medical Center, Chicago, IL 606-12, USA; 4 Molecular Biology Techniques Laboratory, Adam Mickiewicz University, Poznan 61-614, Poland; 5 Department of Cell Biology, Poznan University of Medical Sciences, Poznan 60-806, Poland; 6 Department of Obstetrics and Gynecology, Medical University of Lublin, Lublin 20-081, Poland

**Keywords:** cytosine-adenine repeats, P1 promoter, insulin-like growth factor-1 gene, cervical cancer

## Abstract

High oncogenic risk human papillomaviruses (HPVs) are closely associated with cancer of the cervix. However, HPV infection alone may not be sufficient to cause cervical cancer, and other factors or cofactors may have a cumulative effect on the risk of progression from cervical HPV infection to cancer. The present study investigates the cytosine-adenine (CA) repeat polymorphism in the P1 promoter region of the insulin-like growth factor-1 (IGF-1) gene among cervical precancerous and cancer patients and healthy control females. The association between these polymorphisms, tissue and blood serum levels of IGF-1, and cervical cancer risk and progression is evaluated. The material for analysis consisted of blood cells and postoperative tissues from patients diagnosed with low-grade squamous intraepithelial lesions (L-SILs), high-grade squamous intraepithelial lesions (H-SILs) and invasive cervical cancer (ICC). A polymerase chain reaction amplification and the sequencing of DNA were used for the identification of (CA)_n_ repeats in the IGF-1 P1 region and detection of HPV DNA. The blood serum concentration of IGF was determined by enzyme-linked immunosorbent assay. The identification of the IGF-1 protein in the cervical tissues was performed by immunohistochemical analysis. The range of the length of the CA repeats in the study DNA was 11 to 21. However, the most common allele length and genotype in the control and study patients from serum and tissues was 19 CA repeats and a homozygous genotype of CA19/19. Statistically significant differences in the concentration of IGF-1 in the blood serum were observed between H-SILs and controls, only (p=0.047). However, the concentration of IGF-1 in the group of females with CA19/19, CA19<19 and CA19>19 was significantly higher in the group of patients with H-SIL (P=0.041) and ICC (P=0.048) in comparison with the control group. An association was detected between CA repeat length <19 and/or >19, IGF concentration in blood serum and tissues and the development of cervical cancer.

## Introduction

The development of cancer is considered to be a multistep process dependent on genetic and environmental factors. Cervical cancer develops from precursor lesions of the cervix called squamous intraepithelial lesions (SILs). Progression from normal tissue to invasive cervical cancer (ICC) occurs through a series of increasing grades of SILs; low-grade squamous intraepithelial lesions (L-SILs) and high-grade squamous intraepithelial lesions (H-SILs) (1). Persistent human papillomavirus (HPV) infection is a key factor in this process (2). Little is known about the secondary factors or cofactors associated with the progression from persistent HPV infection to precancerous lesions and invasive cancer. Tumor cells overcome the restriction of replication cycles and regulation of apoptosis observed in normal cells, and essentially become immortal. One of the regulators of apoptosis and proliferation is insulin-like growth factor-1 (IGF-1) (3). IGF-1 is an endocrine and paracrine/autocrine growth factor secreted by numerous tissues. IGF is a complex axis consisting of two ligands (IGF-1/IGF-2), two insulin-like growth factor receptors 1 and 2 (IGFR-1, IGFR-2), six insulin-like growth factor binding proteins (IGFBP-1 to 6), four insulin-like growth factor binding protein-related peptides (IGFBP-rP-1 to 4), insulin and insulin receptor (INSR) (4).

Several studies suggest that the IGF axis may play a significant role in the development of cancer. The role of IGF in cancer susceptibility appears to be multifactorial, and the preponderance of data suggests a slightly increased risk of certain cancers due to the higher activity of the IGF system (5). Conversely, patients with congenital deficiencies in IGF-1 demonstrate a protective effect against cancer development (6). According to Laron *et al* (6), congenital absence of IGF protects against the development and progression of neoplastic processes.

Despite the number of factors that influence IGF-1 levels, it has been estimated that up to 60% of the variability has a genetic basis (7,8). The gene encoding for the human protein IGF-1 is located in the long arm of chromosome 12 (12q22–24.1), covers an area of ~90 kb and contains six exons separated by long (1.9–50 kb) introns. The sequence of the IGF-1 gene is highly conserved, and its transcription is under the control of two promoters, P1 and P2. The P1 promoter regulatory region of the IGF-1 gene is highly polymorphic. It is estimated that approximately 90% of IGF-1 transcripts control P1. The P1 promoter region of the human genome consists of 322 nucleotides located in the 5′UTR of exon 1 and a regulatory region of 1,630 nucleotides. The most conserved is the 322-nucleotide stretch of the 5′UTR. The P1 promoter sequence lacks typical sequences of other genes, such as the TATA or CCAAT element and other areas rich in GC. The P1 promoter has five sections protected from DNase digestion: HS3A, HS3B, HS3C, HS3D and HS3E. HS3D is considered to be responsible for the regulation of the expression of IGF-1 by estrogens (9,10). The position 648 bp of P1 lies (CA)_n_ microsatellite repeat polymorphism comprises a variable length of CA repeat sequence. The number of CA repeats ranges between 10 and 25 with the most common allele containing 19 repeats, characteristic of Caucasians (9,11). The polymorphism of the promoter CA dinucleotide repeats is associated with IGF-1 serum level, birth weight and body height, and with diseases including diabetes, cardiovascular disease and cancer (12–14).

The present study aimed to analyze the association between the CA dinucleotide repeat length polymorphism in the P1 promoter region of the IGF-1 gene in the peripheral blood cells and cervical tissue samples of females with SILs of the uterine cervix and squamous cervical cancer.

## Materials and methods

### Clinical samples

Materials for the evaluation of IGF-1 and CA repeat analysis of the P1 promoter region of the IGF-1 gene comprised of: i) peripheral blood obtained prior to surgery from the antecubital vein of 160 patients enrolled at the gynecological surgery at the Department of Gynecological Oncology and Gynecology, Medical University of Lublin, Poland, and from 67 females voluntarily recruited by doctors and nurses; ii) paraffin-embedded tissue sections obtained from paraffin blocks of patients who underwent surgery at the Department of Gynecological Oncology and Gynecology, Medical University of Lublin, Poland.

Written informed consent was obtained from all the subjects included, and the study was carried out in accordance with the principles of the Helsinki Declaration. The study was approved by the Ethics Committee of the Medical University of Lublin (Resolution of the Bioethics Committee no. KE 0254/263/2011).

The study involved 160 patients with the following postoperative histopathological diagnoses: L-SIL (n=52), H-SIL (n=54) and ICC (n=54). In the ICC group, all 54 patients had squamous cell carcinoma. According to the WHO classification (15), a high degree of differentiation (G1) was observed in 12/54 (22.2%) patients, a moderate degree of observed in 31/54 (57.4%) and a low degree of differentiation in 11/54 (20.4%). According to the FIGO classification (16), 41/54 patients (75.9%) were classified as stage I and 13/54 patients (24.1%) as stage IIa.

The control group consisted of 67 paraffin sections taken from patients referred for diagnostic procedures of the cervix, among which the histopathological results were cervicitis and/or endocervicitis chronica. Serum and tissues (67 samples) were obtained from the same patient. Diagnoses were confirmed or negated by two independent pathologists.

The average age of the patients in the control group was 45±8.2, and in the test group it was 47.2±8.3.

### Determining the level of IGF-1 in plasma by enzyme-linked immunosorbent assay (ELISA)

Concentrations of IGF-1 were determined by ELISA according to the manufacturer’s recommended procedure for the quantitative determination of human IGF-1 concentrations in cell culture supernatants, serum and plasma (cat. no. DG100; R&D Systems, Minneapolis, MN, USA).

### Isolation of DNA from whole blood cells

DNA was isolated from peripheral blood cells using a DNA isolation kit (QIAamp DNA mini kit; cat. no. 51306; Qiagen, Hilden, Germany).

### Isolation of DNA from paraffin sections of tissue

Paraffin blocks with tissue fixed in 10% buffered formalin were cut into two or three sections, 4 μm thick. The microtome was rinsed with alcohol before cutting each block. A new cutting blade was used for the cutting of each of the paraffin blocks. The sections obtained in this manner were placed in a 1.5-ml testing tube with polypropylene and maintained at 4°C for future studies.

The isolation of DNA from archival paraffin tissue was carried out using the Maxwell 16 FFPE Plus LEV DNA Purification kit (cat. no. AS1135; Promega, Madison, WI, USA), apparatus for automated DNA isolation, and a computer program for this kit (cat. no. AS1250; Promega). The resulting DNA was used as a template in a polymerase chain reaction (PCR) reaction for the analysis of CA repeats in the P1 promoter region of the IGF-1 gene.

Quantitative determination of the DNA obtained was carried out by a spectrophotometric method using an automatic spectrophotometer Novaspec II (Pharmacia Co.).

### Identification of HPV DNA

HPV DNA in isolated total DNA was identified by PCR amplification of the HPV gene sequence using the primers MY09, MY11 (17) and LC1, LC2 (18) complementary to the genome sequence of most common types of HPV as described previously (17,18).

### Analysis of CA repeats in P1 region of IGF-1

An analysis of the (CA)_n_ repeats of the IGF-1 gene located 1 kb upstream from the transcription start site was performed using PCR and fragment analysis. PCR was performed in 15-μl volumes consisting of 100 ng genomic DNA, 3.75 pmol fluorescently labeled forward primer (FAM) (5′-AAGAAAACACACTCTGGCAC-3′) and 3.75 pmol reverse primer (5′-ACCACTCTGGGAGAAGGGTA-3′), 0.01 mM deoxy-NTP, 1.5 mM MgCl_2_, 1X PCR buffer and 0.6 units HiFi DNA polymerase (Novazym, Poland; Cat. No. N1003-05). The analysis was performed using a thermal cycler (TGradient Thermocycler, Biometra, Germany). Amplification cycles included one cycle of 4 min at 94°C and 28 PCR cycles consisting of 5 sec at 94°C (denaturation), 30 sec at 60°C (annealing), 1 min at 72°C (elongation), and a final elongation step at 65°C for 30 min. Analysis of the size of the PCR product was carried out on an automated ABI 3130 XL sequencer camera and determined in comparison with the internal GS600LIZ size markers (Applied Biosystems, Inc., Foster City, CA, USA). The estimation of CA repeat numbers in each of the analyzed specimens was based on an extrapolation to the previously developed specific allelic ladder. The ladder marker consisted of 14 sequenced amplifications representing alleles with 7, 9, 11, 13 and 23 CA repeats.

### Immunohistochemical studies of the expression of IGF-1

Immunohistochemical assessment of IGF-1 protein expression was carried out using a the LSAB+System-HRP (rabbit, mouse, goat) kit (DAB+, cat. no. K0679; Dako, Carpinteria, CA, USA). The method used goat polyclonal antibody directed against the human IGF-1 (cat. no. 18 773; Sigma-Aldrich, St. Louis, MO, USA). Antibodies were diluted in antibody diluent with background-reducing components (cat. no. S3022; Dako).

### Semiquantitative scoring of slides

Immunohistochemical evaluation of IGF-1 protein expression was performed independently by two pathologists. Pathologists counted the cells with ×200 magnification in a field of 16 squares, which corresponded to an area of 0.25 mm^2^. The percentage of immunopositive cells was scored according to the method of Nakagawa *et al* (18) as follows: 0, negative staining (<5% stained); +1, weak staining (5–24% stained); +2, moderate staining (25–50% stained); and +3, strong staining (>50% stained).

### Quantitative scoring of slides

The measurement of immunoreactive cells was performed using Cell-2 software, ver. 4.1 (Poznan University of Medical Sciences, Poland). This method is based on an analysis of the distribution of colors and their optical density. The software identifies cells with an optical density greater than the background and on the basis of the color ratio classifies cells as immunoreactive. To determine the percentage of positive cells in the sections, the number of immunopositive cells was divided by the total cell count. A minimum of 5,000 cells was counted in a single section. An investigator who was blinded with regard to the condition of the sample performed all analyses. Statistical analysis of the results was performed with the Kruskal-Wallis test with Dunn’s post-test using Statistica software ver. 5 (StatSoft, Krakow, Poland). P<0.05 was considered to indicate a statistically significant difference in the HC-immunohistochemical studies of the expression of IGF-1 in tissues.

### Statistical analysis

The values of the analyzed parameters were measured using a nominal scale characterized by multiplicity and percentage, while the ratio scale was assessed using the mean, median, standard deviation, lower and upper quartiles, and range of variation. The differences or correlations between the analyzed parameters were verified employing multi-way tables and the homogeneity or independence were tested with the χ^2^ test. Due to the skewed distribution of measurable parameters, evaluated on the basis of the Shapiro-Wilk test, the analysis of the differences between the studied subgroups was performed by non-parametric tests. The comparison of two independent groups was performed using the Mann-Whitney U test. To compare more than two groups, the Kruskal-Wallis test and multiple comparisons/post hoc tests were carried out. The analysis assumed a 5% error of inference and the associated significance level of P<0.05 was used to indicate the existence of statistically significant differences. Statistical analyses were performed with Statistica software ver. 8.0 (StatSoft).

## Results

### Analysis of blood serum IGF-1 level

The IGF-1 blood serum levels for the study and control groups are presented in Table I. The comparative analysis of study groups versus the control group based on the Mann-Whitney U test revealed statistically significant differences in the concentrations of IGF-1 in the group of patients with H-SIL (P=0.047) and cervical cancer compared with controls (Table I).

### Allelic distribution of CA repeats in IGF-1 gene P1 promoter in DNA isolated from serum and tissue samples

DNA from the blood and tissue of study patients was isolated and the correlation between the CA repeats situated in the P1 promoter region of the IGF-1 gene, serum and tissue level of IGF-1 and cervical cancer development was investigated. The IGF-1 genotype distribution in the total cohort and subcategories is shown in Tables II and III. The length range of CA repeats in the study DNA was 11 to 21; however, the most common genotype in the serum and tissues of the control group was homozygote CA19 repeat (74.6 and 89.6%, respectively). These differences were statistically significant (P<0.001). The other frequent genotypes in the control group were CA19/20 (11.9%) and CA18/19 (10.5%). Among the females with precancerous lesions (L-SIL, H-SIL) and cancer (ICC), the homogenous genotype CA19/19 was observed in the DNA from the serum and the tissue in 32.7 and 28.8% of females with L-SIL, respectively, in 24.1 and 11.1% with H-SIL and in 29.6 and 31.5% with ICC. The other most frequent genotypes in females with L-SIL were CA19/21, with a frequency of 28.9 and 23.1% in the serum and tissue, respectively (Table II), and CA19/20 and CA18/19 in the serum of females with H-SIL (37 and 25.9%, respectively). Alleles CA17/19, CA18/19 and CA19/20 were identified among the patients with ICC. All study differences in the number of CA repeats were statistically significant in groups of patients with H-SIL (P<0.001) and ICC (P<0.01) compared with controls. Among the females with H-SIL it was observed that 96.3% (52/54) had one CA19 allele whereas in the ICC group the figure was 68% (37/54).

CA repeats with a length of <19 were classified as short, repeats of length >19 were considered as long, and others as non-19-19 repeats. Statistically significant differences in the number of CA repeat lengths were observed in the group of patients with H-SIL and ICC in comparison with the controls (Table III).

There was no statistically significant difference in the serum concentrations of IGF-1 with CA repeats in the promoter region P1 of IGF-1 gene in the L-SIL patients with 19-19 repeats in comparison with the concentration of IGF in the blood serum of patients with 19>19, 19<19 or non-19-19 repeats in the study groups and the control group (Table III). However, the concentration of IGF-1 in the 19-19 group and the non-19-19, 19>19 and 19<19 group was significantly higher in the H-SIL group (P=0.041) and ICC group (P=0.048) compared with the control group (Table III).

### IGF-1 (CA)_n_ genotype in DNA isolated from the peripheral blood cells and tissues of patients with precancerous and cervical cancer

In an additional study we analyzed the (CA)_n_ repeats in DNA isolated from the blood serum and postoperative tissues from the same patients diagnosed with L-SIL, H-SIL and ICC (Table IV).

The frequency of the homozygote CA19/19 (in blood and tissue) in females with L-SIL was observed to be 21.2% of study patients. This was not statistically significant. Statistically significant differences in CA repeats (in blood and tissue) were observed in females with H-SIL (50.3%) and ICC (50.3%) for the homozygote group CA19/19 (Table IV).

Statistically significant differences in CA repeats were observed in females with H-SIL and ICC between the study and the control groups (Table II). A total of 20% of patients had identical CA repeats in blood and tissue (Table IV). This finding suggests that certain factors for cervical cancer promotion or progression may be dependent on genetic differences in the gene regulation of transcription.

### Analysis of IGF-1 expression in tissues during cervical cancer development

**Figure 1:**
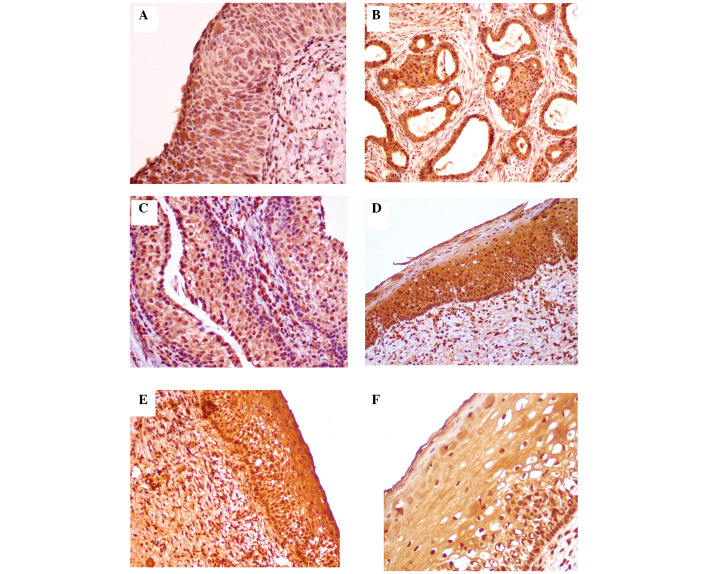
Immunohistochemical analysis of the expression of insulin-like growth factor-1 (IGF-1) in cervical cells in various steps of carcinogenesis. (A) Low-grade squamous intraepithelial lesion; magnification, ×100. Ex+1. (B) HPV infection and high-grade intraepithelial lesion (H-SIL); magnification, ×100. Ex+1. (C) Preinvasive squamous cell carcinoma of the cervix (H-SIL); magnification, ×100. Ex+3. (D) Squamous cell carcinoma of the cervix (G2); magnification, ×100. Ex+3. (E) Cervix and cervical canal; magnification, ×100. The expression of IGF-1 is +1 in parabasal layers of the epithelium, whereas it is negative in the superficial layers of the epithelium. (F) Squamous cell carcinoma (G2) of the cervix, keratinizing type; magnification, ×50. Ex+3.

The IGF-1 expression in tissue samples of precancerous lesions and cervical cancer cases is presented in Fig. 1.

### Semiquantitative scoring of slides

The results of the semiquantitative analysis of IGF-1 protein expression are summarized in Table V.

As demonstrated, IGF-1 protein expression in the L-SIL group was denoted as +3 in 1.9% of slides, +2 in 19.2% of slides, +1 in 69.3% of slides, and in 9.6% there was no expression. In the H-SIL group the results were, respectively, +3 in 44.4%, +2 in 29.6%, +1 in 20.5%, and in 5.5% of samples there was no expression. In the ICC group, positive IGF-1 expression was designated as +2 in 46.3% and +3 in 53.7% of cases. The χ^2^ test revealed a statistically significant association between the histological diagnosis and the percentage of IGF-1 immunopositive cells (P<0.0001).

Additionally, Spearman’s correlation coefficient was evaluated to verify whether the degree of reaction intensity was dependent on the diagnosis. A statistically significant, strong positive correlation between the type of diagnosis and intensity of IGF-1 expression was observed (P<0.05; Table VI).

### Quantitative scoring of the slides

**Figure 2:**
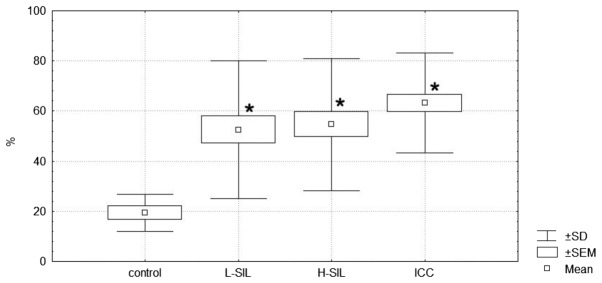
Quantitative scoring of immunohistochemical analysis of IGF-1 expression in cervical cells in various steps of carcinogenesis.^*^p<0.05.

Positive staining was also scored using the software Cell-2 ver. 4.1 according to the percentage of cells with positive staining and type of histopathological diagnosis. The obtained data are expressed graphically as the median and range in Fig. 2.

Statistical analysis revealed that the number of IGF-1-expressing cells is significantly higher in L-SIL, H-SIL and ICC versus the control (Kruskal-Wallis test, P=0.032; Dunn’s post-test, P=0.034, P=0.013 and P=0.001 for L-SIL, H-SIL and ICC, respectively). Between the L-SIL, H-SIL and ICC groups, no statistically significant differences were observed.

## Discussion

Genetic polymorphisms that regulate gene expression are crucial factors accounting for human diversity and disease susceptibility. In a systematic survey of human variations in the cis-regulatory regions, IGF-1 was identified to be one of the 23 genes that demonstrated a consistent allelic imbalance. These findings imply that certain functional elements lie within the IGF-1 regulatory region and contribute to the diversity in IGF-1 concentration among individuals (19).

Epidemiological studies indicate that a high concentration of blood-circulating IGF-1 (endocrine fraction) is a risk factor in numerous types of cancer, including colon cancer (20), prostate cancer (21,22), breast cancer (22) and non-small cell lung cancer (23). Although the level of circulating IGF-1 is affected by various factors, including growth hormones and IGFBP concentration, gender, age, ethnic group and nutritional status (24), studies have demonstrated that 38% of the variance in IGF-1 concentration among individuals is caused by genetic effects (24). Early studies suggested a possible role of the IGF system in cervical carcinogenesis (25).

The key factor in the development of cervical cancer is HPV infection (2). However, the virus alone is not sufficient for the development of cervical cancer, and other factors or cofactors are necessary for the cancer to occur. HPV factors or cofactors in cervical cancer may act by influencing the acquisition of HPV infection, by increasing the risk of HPV persistence or by increasing the risk of progression from HPV infection to H-SIL or ICC. A number of cofactors or factors have also been identified to distinguish ICC from H-SIL or L-SIL (2).

A comparative analysis of IGF-1 in the blood serum and cervical tissues of patients carried out by the authors in this study revealed statistically significant higher levels of IGF-1 in the group of patients with H-SIL. Analysis of IGF-1, depending on the concentrations of its serum level of IGF-1 with repeat CA in the P1 promoter region of the IGF-1 gene for subjects with or without CA19-19 repeats, demonstrated statistically significant higher levels in the H-SIL and ICC groups, compared with others.

The higher concentrations of IGF-1 in H-SIL suggest the involvement of IGF-1 in cervical carcinogenesis induced by HPV. Results of a study by Shen *et al* (25) reveal that IGF-1 may be a potential stimulus for the proliferation and invasion of cervical cancer cells, through effects on integrin ανβ3, which is considered to be one of the possible HPV receptors. According to Bruchim and Werner (26), IGF-1 also stimulates potassium chloride cotransporters, which are required for the invasion and proliferation of cervical, ovarian and breast cancer cells. Additional E6 HPV viral proteins deactivate IGFBP-1, which increases the bioavailability of IGF-1 to IGFR-1 (27). It is also suggested that the oncogenic proteins E6 and E7 of HPV have a distorting effect. In turn, an inhibition of the proliferation of cervical cancer cells may be accomplished by antibodies directed against IGFR-1 (28). An *in vitro* study by Nakamura *et al* (29) indicates the possibility to reverse the phenotype of tumor cell lines by controlling the up-down IGFR.

Prospective studies evaluating the dependence of the natural history of cervical cancer in relation to the IGF axis were performed by Harris *et al* (30) in a group of 137 females. The results of this study confirmed the effect of the IGF system on the development of cervical cancer involving oncogenic types of HPV, and also revealed that a high ratio of IGF-1/IGFBP-3 was associated with an increase in chronic HPV infection (adjusted hazard ratio, −0.14; 95% confidence interval, 0.04–0.57).

Similar correlations have been demonstrated by Lee *et al* (31), who studied serum IGF-1 and IGFBP-3 levels in a group of 44 patients with ICC, 82 patients with cervical intraepithelial neoplasia (CIN) and a control group of 40 patients without neoplastic lesions of the cervix. The average concentration of IGF-1 in the serum of patients with CIN was higher compared with the control group, while for the ratio of IGF-1/IGFBP-3, the decomposition quartile was significantly higher (P=0.041). These data do not support the results of Schaffer *et al* (32), which suggest that IGF-1 may be considered a biomarker of CIN progression.

However, a case study published by Jozefiak *et al* (33) revealed, in turn, significantly lower concentrations of IGF-1 (P<0.001) in the serum of patients with ICC in comparison with the control group. These authors also observed no statistically significant difference between the serum levels of IGF-1 in HPV-positive and HPV-negative individuals.

A meta-analysis of CA repeats in the promoter region of IGF-1 was carried out by Chen *et al* (13). The meta-analysis included 17 studies in a group of 8,799,901 patients and 13,901 controls, seven studies in patients with prostate cancer (2,307 cases and 2,622 controls), seven studies of breast cancer (3,533 cases and 7,771 controls), and three studies of colorectal cancer (2,959 cases and 3,508 controls). The odds ratio (OR) for the CA19 repeat allele versus the non-19 allele was 1.03 (95% confidence interval, 0.95–1.11; P<0.0001). There was no statistical survival depending on both the recessive and dominant modeling CA19 allele. No effect of repetition (CA), 19 patients with breast cancer (seven comparisons: OR=1.03; 95% CI, 0.90–1.17; P=0.005), prostate cancer (seven comparisons: OR=1.08; 95% CI, 0.88–1.27; P=0.0002), and colon cancer (three comparisons: OR=0.96; 95% CI, 0.89–1.03; P=0.36). No evidence was observed that the CA19 allele is associated with cancer risk in Caucasians and Asians. This meta-analysis revealed that repeat polymorphism CA19 is not a major determinant of susceptibility to cancer within the population. However, the authors emphasize the need for large-scale population-based studies to further assess the polymorphisms of IGF-1 CA19 and the assessment of cancer risk in a defined population.

Two published studies have demonstrated that CA19 was significantly associated with a higher level of circulating IGF-1 (34,35). However, the results on this issue were inconsistent, as a different study revealed the opposite association (36) while a further study by DeLellis *et al* (37) revealed no association. In a study by Chen *et al* (38), the results demonstrated that the CA repeat microsatellite by itself was not significantly associated with serum IGF-1 levels. This is consistent with the multiethnic cohort study conducted by DeLellis *et al* (37). One possible explanation for the discrepancy between the present study and other studies is the difference in ethnicity of the study population. The majority of association studies of IGF-1 are based on a Caucasian population, in which the CA19 allele is more prevalent and the genotype and haplotype compositions are different from those of the Chinese population (39).

Studies of CA repeats in the DNA of the P1 promoter of IGF-1 are ambiguous and controversial (38–41) and do not apply to repeat CA gynecological diseases.

Costalonga *et al* (40) hypothesized that IGF-1 is a mediator of growth hormone, and therefore may be considered as a candidate gene for the recombinant human growth factor (rhGH). Adults homozygous for the CA19 repeat in the P1 promoter of IGF-1 had lower serum levels of IGF-1. The aim of this study (40) was to evaluate the effect of CA repeat polymorphisms in the IGF-1 gene in response to the growth of recombinant growth factor therapy in patients with growth hormone deficiency. The authors concluded that the presence of homozygosity for the CA19 IGF-1 gene is associated with less favorable short- and long-term effects of growth following rhGH treatment in patients with severe growth hormone deficiency.

Clinical research by Chen *et al* (38) was a concern for the Chinese population. The researchers defined the haplotype patterns, both SNP and microsatellite repeats, and evaluated their impact on the concentration of IGF-1. This was the first study in which patterns of microsatellite instability haplotypes and SNPs in the P1 promoter of IGF-1 were considered together. The results revealed that microsatellite instability and haplotypes were correlated with the serum concentration of IGF-1 in a group of 450 premenopausal Chinese females.

In the present study, we compared the microsatellite instability of DNA in Caucasian females, obtained from the peripheral blood cells of CA DNA obtained from sections of the same patients stored in surgical archives. The observed microsatellite instability, identical in blood and in tissue, may suggest the nature of hereditary changes in DNA. In contrast, the instability in the blood, which differs from the CA of DNA from tissue, may suggest that postoperative neoplastic changes may be the result of local changes.

This suggests the existence of a specific gene predisposing to tumor genesis, as evidenced by microsatellite instability in the DNA of white blood cells and in tumor tissue.

A search of the available databases for microsatellite instability in DNA promoter P1-IGF-1 did not reveal any studies comparing the repetition of CA in peripheral blood and tissues. Thus, any interpretation of the results is at present extremely difficult and requires execution and repetition of studies in a larger group of patients quantitatively with SILs and ICC.

The transcriptional activity of IGF-1 emphasized the role of the P1 promoter polymorphism of IGF-1 (14). The results of Pacholska-Bogalska *et al* (14), analyzing P1-IGF-1 DNA from blood and tissue cells with CIN and cervical cancer, revealed that a single nucleotide polymorphism at position −383 C>T P1 promoter IGF-1 was present in 16% of HPV-positive patients with precancerous and cancerous changes of the cervix.

The expression of IGF-1 protein in tissue samples was revealed to be statistically significantly higher in precancerous lesions of the cervix and squamous carcinoma of the cervix than in the control group.

In recent years, evidence has been mounting that the IGF axis may be involved in human cancer progression (5) and may be targeted for therapeutic intervention. The current findings in patients with advanced cervical cancer support this. In the future, the association of IGF-1, the IGF system and the 19-19 repeat P1 promoter IGF-1 gene and the clinical outcome of cervical cancer patients in post-treatment samples may add significance in disease mapping as a prognostic marker. It also indicates a possible use in developing newer therapeutic drugs and identifying their targets as well as in the assessment of the clinical outcomes of the disease.

## Figures and Tables

**Table I:** Characteristics of insulin-like growth factor-1 (ng/ml) in blood serum of patients from the study and control groups.

Group	n	Mean	SD	Median	Q1	Q3	Min-max	P-value
Control	67	172.9	81.14	154.6	120.4	201.1	100–414	-
L-SIL	52	183.4	71.45	189.1	148.5	222.4	120–245	0.220
H-SIL	54	210.7	40.89	180.1	156.5	206.1	130–420	0.047
ICC	54	226.1	87.40	186.9	151.1	280.6	110–573	0.090

n, number; SD, standard deviation; Q1, lower quartile; Q3, upper quartile; min-max, minimum-maximum range; L-SIL, low-grade squamous intraepithelial lesions; H-SIL, high-grade squamous intraepithelial lesions; ICC, invasive cervical cancer.

**Table II:** Comparison of evaluation of microsatellite instability (CA repeats) in DNA isolated from peripheral blood cells and paraffin tissues of patients from the study and control groups.

	Control, n (%) (n=67)		L-SIL, n (%) (n=52)		H-SIL, n (%) (n=54)		ICC, n (%) (n=54)	
				
Group	Serum	Tissue	Serum	Tissue	Serum	Tissue	Serum	Tissue
IGF-1 (CA)_n_ genotype								
CA11/19	-	-	2 (3.8)	1 (1.9)	-	-	-	-
CA17/18	-	-	3 (5.7)	3 (5.8)	2 (3.7)	1 (1.9)	-	2 (3.7)
CA17/19	-	2 (3.0)	7 (13.5)	10 (19.2)	-	7 (13.0)	6 (11.1)	7 (13.0)
CA17/21	-	-	-	-	-	-	-	
CA18/19	7 (10.5)	1 (1.5)	4 (7.7)	8 (15.4)	14 (25.9)	7 (13.0)	8 (14.8)	10 (18.5)
CA18/20	-	-	-	-	-	2 (3.7)	-	-
CA18/21	2 (3.0)	1 (1.5)	-	-	-	-	8 (14.8)	2 (3.7)
CA19/19	50 (74.6)	60 (89.6)	17 (32.7)	15 (28.8)	13 (24.1)	6 (11.1)	16 (29.6)	17 (31.5)
CA19/20	8 (11.9)	2 (3.0)	4 (7.7)	-	20 (37.0)	23 (42.6)	8 (14.8)	9 (16.7)
CA19/21	-	1 (1.5)	15 (28.9)	12 (23.1)	5 (9.3)	7 (13.0)	5 (9.3)	6 (11.1)
CA20/20	-		-	3 (5.8)	-	1 (1.9)	3 (5.6)	1 (1.9)
IGF-1 (CA)_n_ genotype groups								
Group 1								
19/19	50 (74.6)	60 (89.6)	17 (32.7)^a^	15 (28.8)	13 (24.1)	6 (11.1)	16 (29.6)	17 (31.5)
19/non-19	15 (22.4)	6 (8.9)	32 (61.5)	34 (65.4)	39 (72.2)	44 (81.5)	35 (64.8)	32 (59.2)
Non-19/non-19	2 (3.0)	1 (1.5)	3 (5.8)	3 (5.8)	2 (3.7)	4 (7.4)	3 (5.6)	5 (9.3)
P-value^a^	<0.001	<0.001	<0.01	<0.01	<0.01	<0.001	0.781	0.341
P-value^b^	0.071		0.059		0.063		0.069	
Group 2								
<CA19	9 (13.4)	4 (5.5)	16 (30.8)	29.0	16 (29.6)	17 (31.5)	22 (40.8)	21 (38.9)
CA19	50 (74.6)	60 (89.5)	17 (32.7)	30.3	13 (24.1)	6 (11.1)	16 (29.6)	17 (31.5)
>CA19	8 (12.0)	3 (5.0)	19 (36.5)	40.7	25 (46.3)	31 (57.4)	16 (29.6)	16 (29.6)
P-value^a^	<0.01	<0.01	0.94	0.872	0.078	0.069	0.89	0.91
P-value^b^	0.070		0.058		0.055		0.064	
Group 3								
CA19 allele present	65 (97.0)	66 (98.5)	49 (94.2)	49 (94.2)	52 (96.3)	50 (92.6)	37 (68.5)	49 (90.7)
CA19 allele absent	2 (3.0)	1 (1.5)	3 (5.8)	3 (5.8)	2 (3.7)	4 (7.4)	17 (31.5)	5 (9.3)
P-value	<0.001	<0.001	<0.001	<0.001	<0.001	<0.001	<0.001	<0.001
P-value	0.068		0.055		0.058		0.070	

L-SIL, low-grade squamous intraepithelial lesions; H-SIL, high-grade squamous intraepithelial lesions; ICC, invasive cervical cancer; IGF-1, insulin-like growth factor-1.

a p-value for comparison of CA repeats in DNA isolated from serum and tissue between control, L-SIL, H-SIL and ICC patient groups.

b p-value for comparison of CA repeats in DNA within control, L-SIL, H-SIL and ICC patient groups between serum and tissue.

**Table III:** Covariate-adjusted mean plasma insulin-like growth factor-1 levels (ng/ml) for subjects with or without 19-19 CA repeats.

	19-19 repeats		CA19<19, CA19>19 and non-19-19 repeats		P-value	
			
Group	n	IGF-1 (SD)	n	IGF-1 (SD)	A	B
Control	50	176.2 (72.2)	17	156.8 (71.3)	0.791	-
L-SIL	17	177.2 (70.2)	35	165.6 (72.3)	0.083	0.69
H-SIL	13	194.4 (54.7)	41	193.9 (61.5)	0.841	0.041
ICC	16	212.6 (50.8)	38	228.4 (58.5)	0.948	0.048

A, P-values for differences between 19-19 and other 19 repeats; B, P-values for differences between H-SIL, ICC and control group. L-SIL, low-grade squamous intraepithelial lesions; H-SIL, high-grade squamous intraepithelial lesions; ICC, invasive cervical cancer; IGF-1, insulin-like growth factor-1; SD, standard deviation.

**Table IV:** Insulin-like growth factor-1 (CA)_n_ genotype in DNA isolated from the peripheral blood cells (B) and tissues (T) of patients with precancerous lesions and cervical cancer.

Group	L-SIL, n (%)(n=52)	H-SIL, n (%)(n=54)	ICC, n (%)(n=54)	Total, n (%)(n=160)	P-value
CA19 same in B and T	11 (21.2)	11 (20.1)	10 (18.3)	32 (20.0)	0.087
In B and T some but other 19-19	18 (34.6)	16 (29.6)	17 (31.4)	51 (31.9)	0.069
In B and T different	23 (44.2)	27 (50.3)	27 (50.3)	77 (48.1)	0.070
P-value	0.078	<0.05	<0.05	<0.065	
P-value	0.075	<0.05	<0.05	0.061	

L-SIL, low-grade squamous intraepithelial lesions; H-SIL, high-grade squamous intraepithelial lesions; ICC, invasive cervical cancer; B, blood cells; T, tissue.

**Table V:** Significance of variables compared with the expression of insulin-like growth factor-1.

	Expression, n (%)					
			
Group	0	+1	+2	+3	Total	Statistical analysis
L-SIL	5 (9.6)	36 (69.3)	10 (19.2)	1 (1.9)	52	χ^2^=76.14
H-SIL	3 (5.5)	11 (20.5)	16 (29.6)	24 (44.4)	54	P<0.0001
ICC	0 (0.0)	0 (0.0)	25 (46.3)	2 (53.7)	54	
Total	8 (5.0)	47 (29.4)	51 (31.9)	54 (33.7)	160	

L-SIL, low-grade squamous intraepithelial lesions; H-SIL, high-grade squamous intraepithelial lesions; ICC, invasive cervical cancer.
